# Supramolecular nanoscale drug-delivery system with ordered structure

**DOI:** 10.1093/nsr/nwz018

**Published:** 2019-02-05

**Authors:** Xin Jin, Lijuan Zhu, Bai Xue, Xinyuan Zhu, Deyue Yan

**Affiliations:** School of Chemistry and Chemical Engineering, State Key Laboratory of Metal Matrix Composites, Shanghai Jiao Tong University, Shanghai 200240, China

**Keywords:** supramolecular chemistry, drug-delivery system, host–guest recognition, self-delivery system, precision medicine

## Abstract

Supramolecular chemistry provides a means to integrate multi-type molecules leading to a dynamic organization. The study of functional nanoscale drug-delivery systems based on supramolecular interactions is a recent trend. Much work has focused on the design of supramolecular building blocks and the engineering of supramolecular integration, with the goal of optimized delivery behavior and enhanced therapeutic effect. This review introduces recent advances in supramolecular designs of nanoscale drug delivery. Supramolecular affinity can act as a main driving force either in the self-assembly of carriers or in the loading of drugs. It is also possible to employ strong recognitions to achieve self-delivery of drugs. Due to dynamic controllable drug-release properties, the supramolecular nanoscale drug-delivery system provides a promising platform for precision medicine.

## INTRODUCTION

Supramolecular chemistry engineers multiple non-covalent interactions together that gives rise to charming properties, including specificity, reversibility and tunability [1–3]. Although supramolecular affinities (including hydrogen bonding, metal chelation, hydrophobic interactions, π–π stacking and van der Waals interaction) are usually weak, most of them are capable of providing specific recognition on a molecular level [4–7]. Derived from the transient nature as well as molecular recognition, supramolecular chemistry has drawn great research focus over the past three decades and relative research won the Nobel Prize in 1987 and 2016 [8,9].

Supramolecular affinity is also crucial in bio-environments. Multiple supramolecular interactions co-exist within biological systems as a result of a delicate balance, resulting in the ordered and hierarchical structure and function of living organisms [10,11]. Inspired by nature systems, supramolecular interaction has been widely utilized in the biomedical field, such as in the construction of nanoscale drug-delivery systems [12,13]. The nanoscale drug-delivery system has been regarded as a preferable drug-administration strategy compared to free drugs [14]. Nano-carriers have been proven to not only improve drug solubility, but also limit off-site drug accumulation [15,16]. Moreover, the supramolecular design could incorporate some unique properties into the nanoscale drug-delivery system, such as molecular-level design and dynamic/recoverable properties, resulting in a feasible and novel drug-delivery strategy [17].

A large amount of research utilizing supramolecular affinity for nanoscale drug-delivery systems has been witnessed [18–23]. Our review focuses on the specific and highly organized drug-delivery systems designed with clear interacting mechanisms, such as host–guest interactions, polyvalent hydrogen bonding and nucleic acid base complementary pairing. Ordered nano-structures through metal coordination are covered in a large series of research that has been extensively reviewed elsewhere, so we exclude this part from our review. In discussed research, supramolecular interaction might play a dominant role either in delivery-carrier formation or in the therapeutic-loading process. In a typical nanoscale drug-delivery system, the carrier materials and therapeutic payloads are essential components, and there might be different principles for supramolecular design for nano-vehicles and loading methods. Therefore, we group the research work into (i) supramolecular drug carriers, (ii) supramolecular interaction-mediated drug loadings and (iii) supramolecular self-delivery systems, and review this work in different sections independently. In the next section (on supramolecular drug carriers), we focus on how to employ supramolecular interactions as the main driving force in building nanoscale drug carriers. In the third section, we introduce how supramolecular interaction has been used as the main linkage between vehicles and therapeutics. In the fourth section, we discuss a novel supramolecular delivery strategy for the therapeutics-only system, in which nanoscale delivery could be achieved through supramolecular interaction without carrier materials involved.

## SUPRAMOLECULAR DRUG CARRIERS

A major demand on drug carriers is ‘smartness’, because diseases are always accompanied by local changes in pH, free radicals, over-expressed biomarkers or high concentrations of reactive oxygen species [24–27], so an ideal drug carrier should be able to respond to one or several of these changes in a rapid manner. Supramolecular nanoscale drug carriers have precisely designed chemical compositions and can offer engineered drug-release profiles after drug loading. Benefiting from the nature of non-covalent linkage, supramolecular nano-carriers are in a dynamic/reversible state so that a small change in the environment (including pH, ionic strength, temperature and oxidation) is sufficient to induce a dramatic response [28]. This unique feature makes them ‘smart’ therapeutic vehicles, and it is relatively easy for supramolecular nano-vehicles to achieve a disease-related triggered release of drug payloads, resulting in enhanced therapeutic specificity. At the same time, the balance of supramolecular systems is easily degraded under physiological conditions, leading to a fatal stability problem with unexpected loading exposure [29]. It is necessary for supramolecular-based delivery carriers to possess appropriate bio-stability, in order to overcome obstacles both *in vitro* and *in vivo* [30]. A supramolecular drug carrier is a balance between reliability and smartness, which can only be achieved though rational designs of the supramolecular building block. As most drug carriers are required to be hydrophilic, design principles with different supramolecular building blocks vary greatly. In the next section, we will focus on the design and function of nano-carriers based on supramolecular interaction, introducing several building approaches based on different types of supramolecular building blocks.

### Polymeric supramolecular carriers

Supramolecular affinity can be utilized to construct polymer systems through the rational design of small-molecule units or macromolecule units. Acting as an extra and reliable linkage, supramolecular interaction can drive the units into an ordered and pre-designed architecture with different topologies, including linear chains [31–36], cross-linked networks [5], dendrimers [37,38], hyperbranched polymers [39–45], brushed polymers [46,47] and star polymers [48–54]. Benefiting from the dynamic nature of non-covalent interaction, supramolecularly engineered polymers usually respond to environmental change in a fast and precise way, which makes them good platforms for drug-delivery carriers. To date, various designs of supramolecular polymeric delivery carriers have been reported to achieve the successful delivery of chemo-drugs [55–58], therapeutic enzymes [59–61] and genetic materials [62–65].

**Figure 1.**
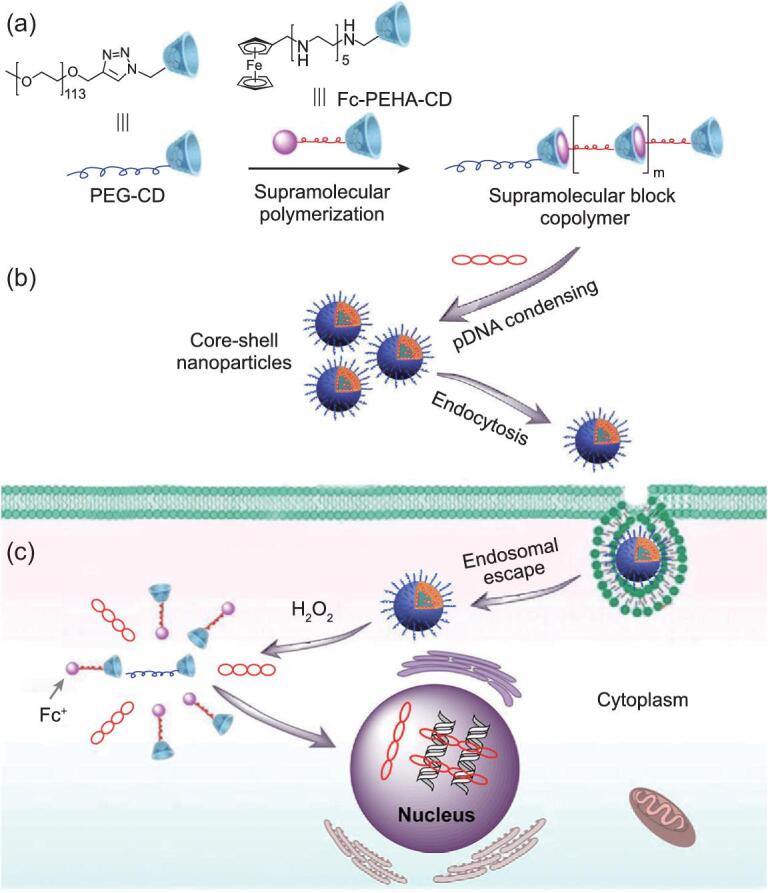
Schematic illustration of (a) supramolecular polymerization route of cationic supramolecular block copolymer, (b) pDNA condensing, and (c) intracellular delivery and H_2_O_2_-triggered release of pDNA *in vitro*. Adapted with permission from [67].

In order to exhibit macromolecular behavior in a supramolecular polymer system, interactions with a high association constant (like host–guest recognition) are usually employed for maintaining a relatively large molecular weight [66]. The responsiveness of the carrier depends on the specific properties of the building blocks as well as the association constant of the supramolecular domain. Wumaier *et al.* designed a supramolecular plasmid DNA (pDNA) vector consisting of a cationic supramolecular block polymer with two building blocks: β-cyclodextrin conjugated polyethylene glycol (PEG-CD) and ferrocene conjugated pentaethylenehexamine with CD modification (Fc-PEHA-CD) (Fig. 1) [67]. The binding ability of such a supramolecular vector was achieved through the multivalent electrostatic interaction between DNA and PEHA blocks, forming a condensed genetic inner core protected from the PEG blocks. The resultant core–shell nanoparticle exhibited enhanced bio-stability and a high intracellular delivery capacity of DNA. Attributed from the reversible association and disassociation between β-CD and Fc, it also exhibited a fast-release pDNA under an H_2_O_2_ trigger in cancer cells.

### Peptide supramolecular carriers

**Figure 2.**
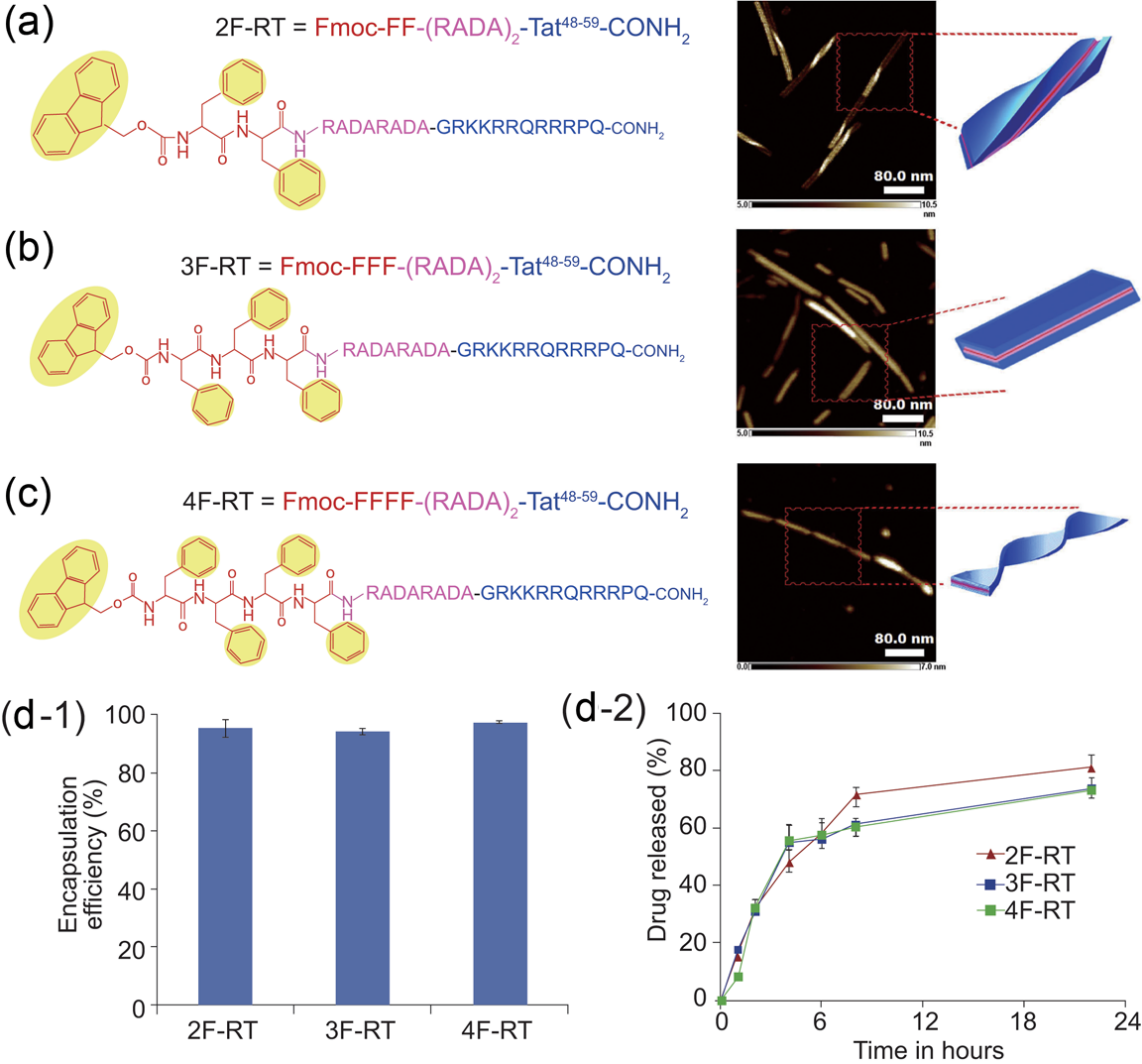
Structure illustration and assembly visualization (AFM tapping image and simulation illustration) of three designs of the Tat-RADA-Fn sequence: 2F-RT (a), 3F-RT (b) and 4F-RT (c). Encapsulation efficiency of autophagy inducer rapamycin (d-1) and the drug-release profile (d-2). Adapted with permission from [73].

Rationally designed amphiphilic peptides, which can form β-sheeted assembles and π–π interaction, are usually utilized for supramolecular assembly in order to obtain ordered nano-vehicles, such as fibers, sheets and ribbons [68–72]. A new example has employed Tat peptides conjugating with a RADA sequence to obtain supramolecularly assembled nanodrills for the intracellular delivery of hydrophobic therapeutics (Fig. 2) [73]. In this design, the cell-penetrating peptide (CPP) Tat derived from HIV1 donates a hydrophobic sequence, providing a high capacity for biological membrane penetration [74]; a RADA sequence modified with various phenylalanine (Phe, F) residues provides a supramolecular complex driving force with a β-sheet formation and π–π stacking. Relying on the hydrophobic region on such a nanodrill, hydrophobic therapeutics such as rapamycin can be easily encapsulated. Benefiting from Tat segments on the surface, the effective intracellular and induction of autophagy can be achieved in selected cell lines.

### Supramolecular liposome

**Figure 3.**
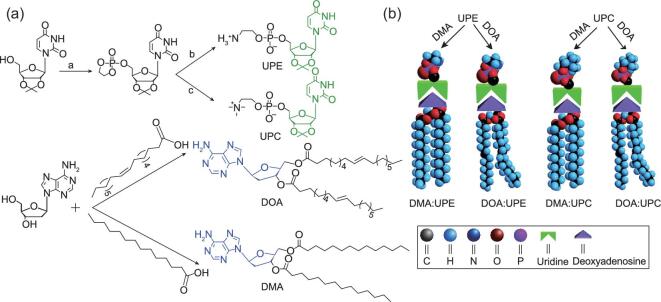
(a) Synthetic route, chemical structure of nucleoside phospholipids and (b) the schematic representation of the formation of supramolecular phospholipids. Adapted with permission from [77].

The release efficiency in conventional liposomes has been regarded as a great challenge in clinical application. Although various stimuli-responsive liposomes have been developed, research on novel liposome systems with optimized controlled drug-release behavior is still in great demand. Differently from covalently bonded liposomes, supramolecularly engineered liposomes might have specialties, such as a fast response to exteriors and effective release of cargos. Inspired by the complementary interaction of base parings in natural DNA and RNA, Wang *et al.* have introduced such an interaction into a novel phospholipid system (Fig. 3). Base pairings are moderately strong, with specific direction resulting from the multivalent hydrogen bonding between adenine–uracil (A–U), adenine–thymine (A–T) and guanine–cytosine (G–C) [75,76]. In their work, hydrophilic head and hydrophobic tails of phospholipids are conjugated with a pair of nucleobases, respectively (Fig. 3b) [77]. These new types of phospholipid with nucleosides could marry each other via base-pairing recognition through a simple mixing procedure and self-assemble into liposome-like bi-layer nano-vehicles. Benefiting from the high sensitivity and acidic pH responsiveness, the supramolecular liposome, after drug loading, exhibited enhanced anticancer ability *in vitro* and *in vivo*. An additional functional benefit of this new generation of liposomes is that multiple choices of nucleobase pairs are available and a variety of phospholipid systems could be designed and easily obtained, depending on different therapeutic demands.

The design and construction of supramolecular nano-carriers are sparkled with wit and charm. Such carriers are also versatile for a variety of drug-loading strategies, obtaining optimized controlled release of drugs, among which passive drug encapsulation and covalent conjugation of drug molecules are used the most [78,79]. For the passive encapsulation mode, the encapsulation is realized through high hydrophobicity from drug molecules and the hydrophobic domain in supramolecular nano-vehicles. The slow drug-release rate is realized through the physical barrier from vehicle materials and could be further regulated through supramolecular affinity change. For covalent drug conjugation, most loaded drugs are well protected and in an inactive state until the liable linkage ruptures. The release rate of conjugated drugs is determined by the rate of bond cleavage.

## SUPRAMOLECULAR INTERACTION-MEDIATED DRUG LOADING

Besides the passive encapsulation and covalent conjugation mentioned above, supramolecular interactions can also act as the main force between drugs and carriers, where non-covalent and specific affinity will govern the drug-loading/release behavior. A significant benefit of introducing supramolecular interaction into the drug-loading process is that the formation and the properties of delivery systems could be highly dependent on the drug itself. An additional functional benefit is realized in the ease of the stimuli-responsive drug release [80]. A large variety of therapeutics can be used as supramolecular building blocks. Most chemo-drugs owned incorporate a planar aromatic structure, such as CPT, PTX and so on; a large number of protein therapeutics show specific binding affinity to certain ligands; and other drugs belong to cytotoxic nucleoside analogs, such as floxuridine. All these structures offer strong affinities to govern a reliable supramolecular drug-loading procedure.

### Host–guest recognition-mediated drug loading

The delivery and release of drugs, in either formulations or bodies, usually occur in an aqueous environment, where the hydrophobic domain on the drug structure can act as a good guest candidate for host–guest recognition in a drug-loading procedure [81,82]. A typical guest drug has a highly hydrophobic structure, which acts as a proton-donating agent to form multiple hydrogen bonds with the proton-accepting structure of the host molecules [83]. Such a supramolecular complex achieves molecular-level protection from drug degradation or deactivation.

A host–guest interaction with a high association constant provides many opportunities for hydrophobic drug loading. Macrocycles, such as cyclodextran (CD), cucurbituril (CB) and crown ester families of macrocycles, are the most popular host candidates to marry drug guests [84,85]. Besides small hydrophobic drugs, a variety of therapeutics can act as recognition guests directly, including certain proteins with N-terminal aromatic amino acids such as insulin and human growth hormone [86,87]. There is no doubt that, after marring with macrocycle hosts, hydrophobic drugs could achieve enhanced solubility and stability [88,89]. Some published literatures have further demonstrated that the host motif could also contribute a positive effect to permeability through biological membranes, and might be a promising platform for oral administration, topical cream, eye drops and nasal sprays [90–92].

**Figure 4.**
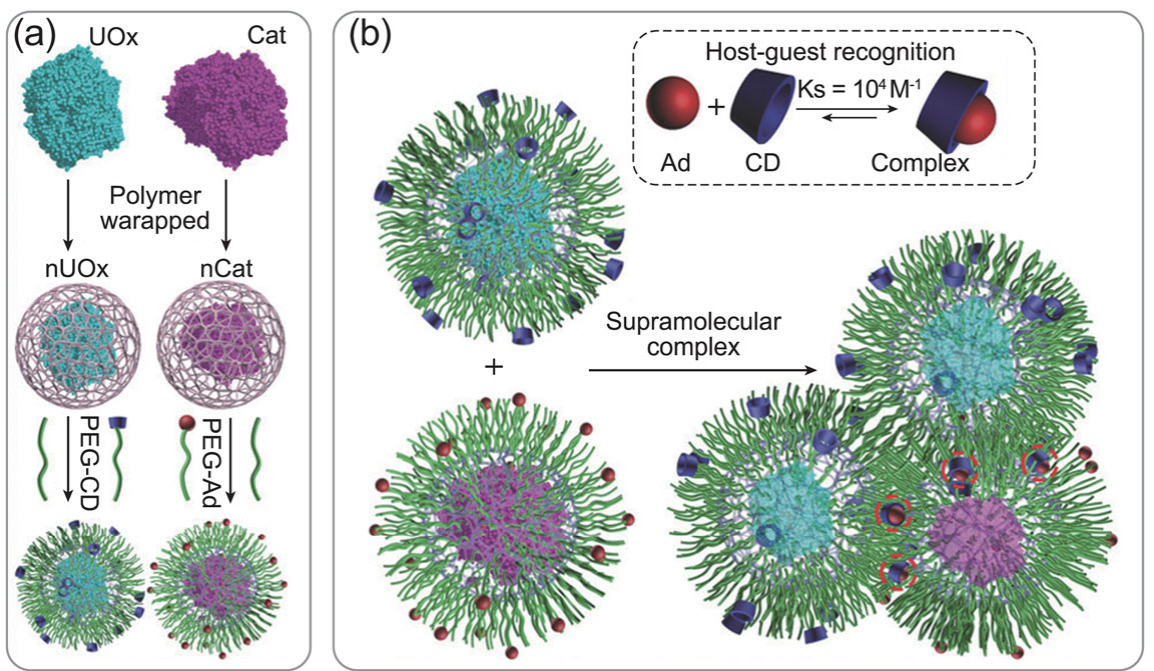
Cartoon illustration of the synthesis of the host–guest-mediated multi-enzyme delivery system. (a) Synthesis route of host building block PEG-CD and guest building block PEG-AD; (b) formation of multi-enzyme nanocluster through host–guest recognition-driven self-assembly. Adapted with permission from [95].

Enzymes are rising stars in disease treatment, as they have a clear mechanism and high therapeutic effect. As a macromolecule with a sophisticated structure, the delivery of enzymatic therapeutics is always of great difficulty. Moreover, two or more enzymes might be in need of cooperation in order to treat a symptom for most diseases [93,94]. Liu and coworkers utilized host–guest affinity to integrate uricase (UOx) and catalase (Cat) dynamically, resulting in a feasible and effective serum uric acid (sUA) control therapy with low systematic toxicity [95]. In their study, CD and adamantine (Ad) worked as a host–guest pair and were conjugated to UOx and Cat through a PEG linker, respectively (Fig. 4a). A multi-enzyme nanocluster with a certain UOx/Cat ratio could be achieved (Fig. 4b). Teamwork could be observed *in vitro* and *in vivo* with fast clearance of sUA levels that resulted from the UOx and reduced H_2_O_2_-induced abnormal cell apoptosis resulted from the Cat. This work employed a supramolecular engineering strategy for the synergism of two enzymes in a nanoscale space, promising a liable and facile platform for multi-enzyme therapeutic systems.

### Base-pairing-mediated drug loading

Nucleoside analog prodrugs, such as clofarabine and floxuridine, have been regarded as types of effective and safe therapeutics for various diseases. An additional benefit from such drugs is that they can work as guest molecules to form moderately strong and directional supramolecular interaction with nucleic acid hosts through the base-pairing principle [96]. A typical nucleoside prodrug loading strategy relies on the rational design of nano-vehicles with engineered DNA strands. Through the supramolecular recognition between engineered DNA strands and nucleoside analogs, both nucleoside analog therapeutics and nucleoside-modified targeted ligands can be integrated on nano-vehicles to obtain multifunctional nanoscale delivery systems [71]. The major reason for using DNA strands instead of a single nucleoside base as supramolecular building blocks is to provide a possibility for tunable binding affinity and a programmable recognition region, which might be crucial to control drug-release profiles.

**Figure 5.**
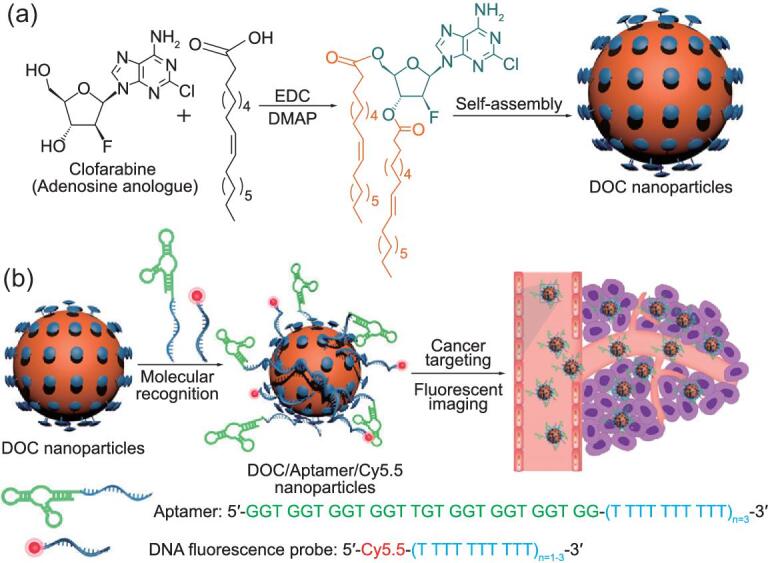
Schematic illustration of (a) the synthesis route and self-assembly of a DOC nanoparticle, and (b) aptamer and imaging agent loading through base-pairing interactions. Adapted with permission from [97].

Wang *et al.* utilized such a strategy in an opposite way, realizing an all-in-one delivery system combining targeting, therapeutic and detective abilities [97]. In detail, they prepared a nanoparticle using (i) a modified nucleoside analog prodrug 3′,5′-dioleoyl clofarabine (DOC), (ii) DNA strands T30 (30-mer poly dT pligonucleotide) modified aptamer with high targeted affinity and (iii) T30 modified imaging probe Cy5.5 (Fig. 5). Based on the supramolecular affinity between clofarabine with T30, this nanoscale delivery platform was obtained in a simple procedure of mixing three components, achieving multiple functions with active tumor-targeting capacity, *in vivo* imaging of the tumor site as well as controlled release of clofarabine. A significant strength of the loading affinity can be programmed with regulation of the DNA-strand sequence.

**Figure 6.**
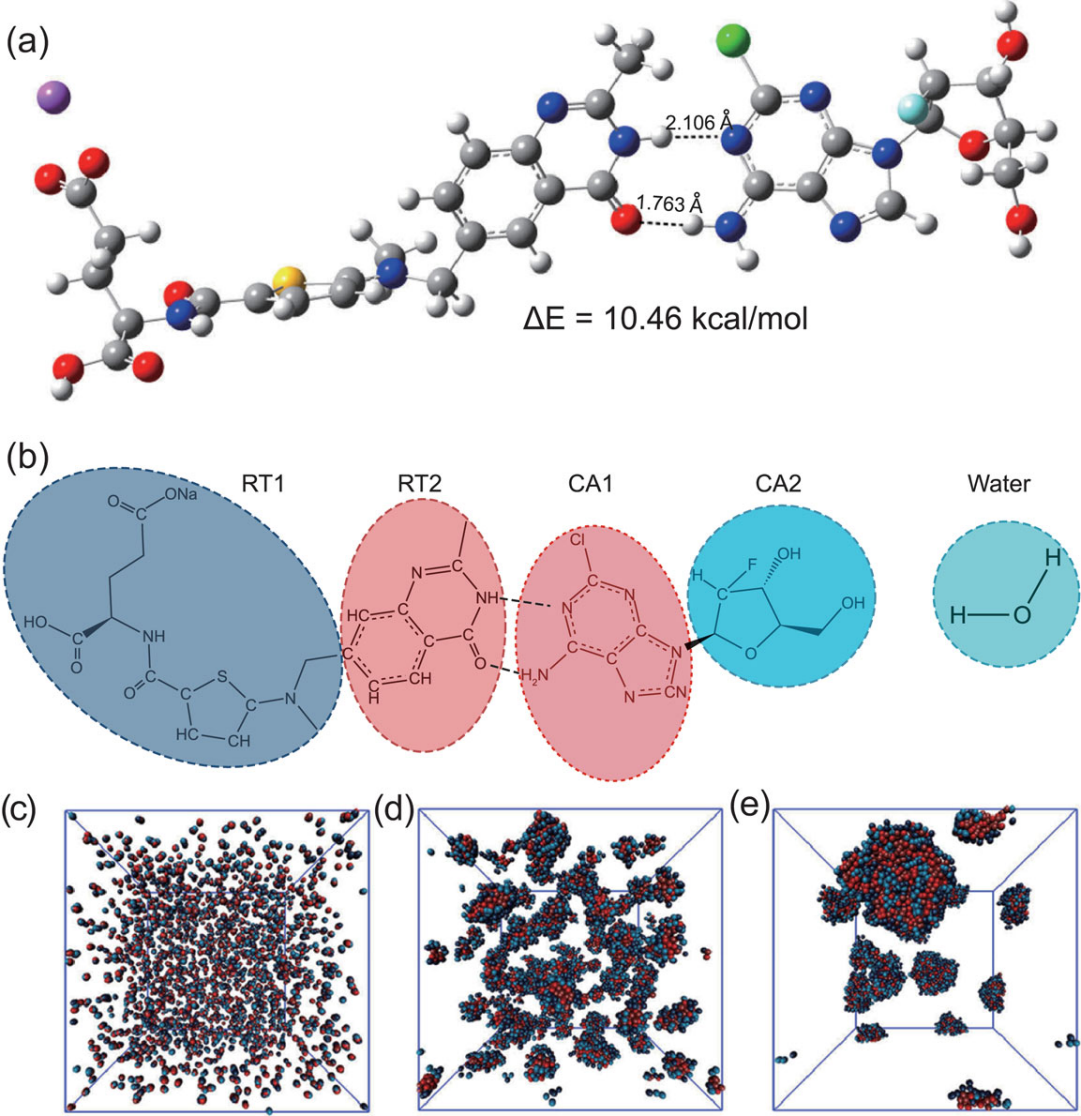
Molecular simulation for the supramolecular interaction and self-assembly of CA and RT in the existence of water. (a) Optimized structure of CA/RT motif with binding affinity calculated using the DFT method; (b) coarse-grained models of CA, RT and water; (c)–(e) DPD simulations of the self-assembly of the CA/RT motif in solution. Adapted with permission from [105].

## SUPRAMOLECULAR SELF-DELIVERY SYSTEMS

A recent trend in nanoscale drug-delivery systems is to develop a self-delivery system with anticancer drugs only, which is able to form the nanoscale delivery of drugs to achieve enhanced therapeutic effects without any help from the carrier [98–100]. Most reported self-delivery nanoscale drug-delivery systems are formed through directly conjugating a hydrophobic drug with a hydrophilic one, in order to obtain an amphiphilic structure. Proven by several pieces of pilot research, the concept of the self-delivery of drugs has been well accepted and highly praised as a promising drug-delivery platform. One concern in such a system lies in the potential structural damage to drugs during the chemical reaction, and replacing the chemical bond with a non-covalent interaction may solve such a problem. Certain supramolecular affinities, such as host–guest interaction and multivalent hydrogen bonding, can mediate the fusion of a hydrophobic domain with a hydrophilic one to form supramolecular amphiphile, which is capable to self-assembly into nanoparticles or nanogels. If the drug structure in such supramolecular amphiphiles is made good use of, with one hydrophobic drug and a hydrophilic therapeutic, a supramolecular self-delivery nanodrug-delivery system can be feasibly accessed [101–103]. Hydrophilic chemo-drugs and peptide therapeutics usually allow the hydrophilic domain to cooperate with hydrophobic therapeutics forming nanoscale delivery systems [104]. The interactions between the two drugs should be in a clear and specific way, in order to obtain ordered nano-assembly and therapeutic function (Fig. 6) [105].

Wang *et al.* integrated two Food and Drug Administration (FDA)-approved drugs (clofarabine as the hydrophilic domain and raltitrexed as the hydrophilic domain) together through molecular recognition [106]. A clear interaction manner happened between the two drugs, which was confirmed by ^1^H NMR and molecular simulation. Such an amphiphilic structure that relies on supramolecular interaction self-assembled into stable nanoparticles in an aqueous system, which exhibited enhanced therapeutic effect *in vitro* and *in vivo*.

**Figure 7.**
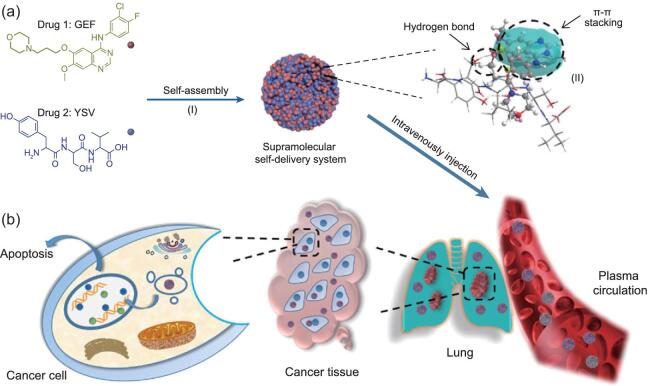
Schematic illustration of the construction and function of the supramolecular self-delivery system. (a) The structure of the drug building blocks and the formation of the supramolecular self-delivery system through self-assembly (I), and simulation illustration of supramolecular affinity between two drugs (II). (b) Delivery behavior and therapy mechanism *in vivo* after intravenous injection. Adapted with permission from [102].

Deng and coworkers employed a peptide therapeutic donated as a hydrophilic domain cooperating with a hydrophobic chemo-drug through multiple hydrogen bonding and π–π interaction (Fig. 7a) [102]. The resultant nano-assembly responded quickly to an acidic environment such as a tumor microenvironment, which might accelerate the rupture of supramolecular linkage and result in a tumor-specific fast drug release. For *in vivo* study, a prolonged retention time in plasma as well as enhanced accumulation in a tumor site were observed after intravenous injection, both of which are typical properties for nanoscale systems (Fig. 7b). Based on these phenomena, we can conclude that supramolecular self-delivery systems could be reliable and stable enough for *in vivo* use after rational design.

## CONCLUSION AND PERSPECTIVE

Supramolecular chemistry has been providing guiding principles for us to understand how nature survives and thrives. What is more, it provides innovative approaches in medicine design. The supramolecular design of drug-delivery system routes from molecule-level building blocks as well as a delicately engineered systems possesses desirable delivery capacity with predictability, precision and limited off-site effects. Additional benefit is realized in the ease of preparation of the supramolecular systems as a ‘mix and match’ procedure. Combining advantages of both supramolecular chemistry and the nano-size effect, better mimicking of bio-functions through simple and effective supramolecular nanoscale drug-delivery systems could be achieved with huge translational benefits.

As supramolecular affinities have made great efforts in nano-carrier design and fancy drug-loading strategies, the supramolecular nanoscale drug-delivery strategy has entered into a new self-delivery era. The disuse of carrier material endows supramolecular drug delivery with feasible formulation and enhanced safety. Additionally, the interaction in such a system mainly happens between two small molecules and the molecular simulation results are usually in high accordance with experimental data. The computational approach to design predicts a novel supramolecular self-delivery system with a high degree of accuracy and productivity in the near future. Compared to conjugated drug-delivery systems, the bio-stability of supramolecular systems is still a concern for clinical use. Innovative solutions for highly stable supramolecular nanodrug-delivery systems are always in demand. A traditional idea for putative stability is mostly based on chemical intuition. However, natural supramolecular systems usually show proper stability and can give us inspiration. On the one hand, we might mimic nature to develop stable supramolecular systems with multiple levels of structural design. In the other, we are looking forward to more computational simulation research based on natural systems to inspire new supramolecular systems.

Precision medicine, aimed at patient-specific medical care, has been recently a hot topic in the pursuit of safe and effective disease treatment or prevention [107]. A novel drug-delivery strategy may offer useful solutions in the content of precision medicine. We therefore believe that supramolecular designs in nanoscale drug delivery, with easy construction, enhanced therapeutic effect and customized treatment options, should provide possibilities and excitement.
